# Preventing Acute Kidney Injury: a qualitative study exploring ‘sick day rules’ implementation in primary care

**DOI:** 10.1186/s12875-016-0480-5

**Published:** 2016-07-22

**Authors:** Rebecca L. Morris, Darren Ashcroft, Denham Phipps, Peter Bower, Donal O’Donoghue, Paul Roderick, Sarah Harding, Andrew Lewington, Thomas Blakeman

**Affiliations:** NIHR School for Primary Care Research, Centre for Primary Care, University of Manchester, 5th Floor Williamson Building, Oxford Road, M13 9PL Manchester, UK; NIHR Greater Manchester Primary Care Patient Safety Translational Research Centre, Institute for Population Health, University of Manchester, Manchester, UK; Centre for Pharmacoepidemiology and Drug Safety, Manchester Pharmacy School, University of Manchester, Manchester, UK; Department of Renal Medicine, Salford Royal NHS Foundation Trust, Salford, UK; Faculty of Medicine, University of Southampton, Southampton, UK; Park Edge Practice, Leeds, UK; Department of Renal Medicine, Lincoln Wing, St James’s University Hospital, Beckett Street, Leeds, UK; NIHR Collaboration for Leadership in Applied Health Research and Care Greater Manchester, Manchester, UK

**Keywords:** Acute Kidney Injury, UK, Primary care, Normalisation process theory, Kidney disease, Qualitative, Multimorbidity

## Abstract

**Background:**

In response to growing demand for urgent care services there is a need to implement more effective strategies in primary care to support patients with complex care needs. Improving primary care management of kidney health through the implementation of ‘sick day rules’ (i.e. temporary cessation of medicines) to prevent Acute Kidney Injury (AKI) has the potential to address a major patient safety issue and reduce unplanned hospital admissions. The aim of this study is to examine processes that may enable or constrain the implementation of ‘sick day rules’ for AKI prevention into routine care delivery in primary care.

**Methods:**

Forty semi-structured interviews were conducted with patients with stage 3 chronic kidney disease and purposefully sampled, general practitioners, practice nurses and community pharmacists who either had, or had not, implemented a ‘sick day rule’. Normalisation Process Theory was used as a framework for data collection and analysis.

**Results:**

Participants tended to express initial enthusiasm for sick day rules to prevent AKI, which fitted with the delivery of comprehensive care. However, interest tended to diminish with consideration of factors influencing their implementation. These included engagement within and across services; consistency of clinical message; and resources available for implementation. Participants identified that supporting patients with multiple conditions, particularly with chronic heart failure, made tailoring initiatives complex.

**Conclusions:**

Implementation of AKI initiatives into routine practice requires appropriate resourcing as well as training support for both patients and clinicians tailored at a local level to support system redesign.

**Electronic supplementary material:**

The online version of this article (doi:10.1186/s12875-016-0480-5) contains supplementary material, which is available to authorized users.

## Background

Acute Kidney Injury (AKI) is a major patient safety issue [1]. It is a clinical syndrome characterised by rapid reduction in kidney function [2]. Globally, AKI affects approximately 13.3 million people per year despite the fact that it is mostly preventable with timely intervention [3]. In the UK, it is estimated to affect between 12 and 14 % of all hospital admissions [4], with almost two thirds of patients having developed it in community settings [5]. AKI is associated with longer lengths of stay and increased requirement for renal replacement therapy [6]. Recently, it has been estimated that AKI is associated with over 40,000 inpatient deaths in England and the annual cost is estimated at £1.02 billion, or 1 % of the NHS annual budget [4]. From 2010 to 2011, the cost of caring for patients with AKI post hospital discharge was £190 million, thus there is a need to prevent, or reduce, the occurrence of AKI [4, 7]. Optimal care including a focus on targeting interventions in primary care has the potential to save up to 12,000 lives per year and produce substantial savings for patients and the NHS [4, 8].

Within NHS England’s patient safety domain, the national Think Kidney programme has been established to tackle harm associated with AKI [9]. In parallel, the growth in demand for urgent care services increases annually and this trend is expected to continue as people live for longer and have more complex care needs [10]. Key measures to address current demands include a more responsive urgent care service outside of hospital with a need for the provision of better support for people to self-care [10]. An underpinning component of high quality care is self-management support [11]. The recent Keogh review highlights the need to implement better self-management options including care planning to help patients ‘deal with their own condition before it deteriorates or additional help is required’ [10].

The prevention of AKI is one pathway to reducing demands on urgent care. Certain patient populations are particularly at risk of complications associated with AKI during episodes of acute illness (e.g. sepsis caused by gastroenteritis). These include patients with diabetes, heart disease, cancer, pre-existing reduced kidney function (chronic kidney disease (CKD)) as well as patients with cognitive decline who are reliant on carer support [2, 3, 12]. In order to prevent AKI, the National Institute of Health and Care Excellence (NICE) recommended the need to communicate risk of AKI with patients, including the need to maintain fluid balance and consider temporary cessation of certain medicines (including angiotensin-converting-enzyme (ACE) Inhibitors) during episodes of acute illness [4, 12, 13]. Resources to support medicines self-management during acute illness have been termed ‘sick day rules’ or sick day rule guidance [14, 15]. The planning for changes to medication regimes during acute illness already occurs for other conditions, in particular sick day rules for type 1 diabetes [14], where patients temporarily adjust their medicine regime during an acute illness for a short period and then restart them. However, although recommended, currently there remains limited evidence surrounding their implementation and effectiveness. In the context of AKI being viewed as a key clinical priority for improving patient safety and outcomes [6, 9, 16], our study aimed to identify factors that enable or constrain the implementation of sick day rules in primary care and the safe reintroduction of temporarily suspended medicines.

## Methods

### Design

A qualitative study design was adopted and Normalisation Process Theory (NPT) was used as a sensitising concept for data collection and analysis [17, 18]. NPT is a theory of healthcare implementation and offers a structure for understanding practises that enable or constrain the integration of an intervention (such as ‘sick day rules’) into routine care [17, 18]. NPT focuses on the work that different actors (e.g. GPs, nurses or pharmacists) have to accomplish to implement the new intervention into practice [18].

### Sampling

We purposively sampled participants (General Practitioners (GPs), practice nurses and community pharmacists) from primary care sites who had experience of implementing a sick day rule plan to prevent AKI in two geographical locations as well as participants from a site who had no experience of using sick day rules. Participants were also purposefully sampled in terms of a range of professions. Clinicians were recruited through snowball sampling using existing contacts, the National Institute for Health Research Clinical Research Network and the Royal Pharmaceutical Society and were sent the study information sheet. For practices that had not implemented a sick day rule plan, a prototype plan developed in conjunction with patient and public involvement partners was discussed (see Additional file 1). Practices that had implemented a sick day rule plan had access to a range of tools: i. a patient leaflet; ii. enhanced laboratory co-ordination; iii. credit card sized patient reminder cards; iv. improved access to a local nephrology team; v. website for professional reference; vi. health professional education. The plan developed as part of this project (see Additional file 1) built on the discussions during the interviews but was not the same as the plan used in these existing sites (for more information on the development of this initiative see http://heeminnovation.co.uk/projects/improving-the-outcome-of-aki-in-primary-care/).

In recognition of guidance that patients with CKD are at increased risk [2, 12], we used a convenience sample of patients who had a known diagnosis of stage 3 CKD and were prescribed either an ACE inhibitor or Angiotensin Receptor Blocker (ARB). Patients were screened to ensure they met the study inclusion criteria and those who were eligible were sent the patient information sheet from their General Practice.

### Data collection

Forty semi-structured interviews were conducted with all participants by RM. Interviews were conducted between May to September, 2014. Interviews were conducted either at participants’ surgeries, community pharmacies or homes. Interviews ranged from 14 to 107 min (average 40 min). The interviews were digitally recorded and professionally transcribed. Interviews explored the management of acute episodes of illness as well as the work required to implement ‘sick day rules’ into everyday practice. This included exploring, the work of embedding and integrating ‘sick day rules’ into: 1) clinical encounters; 2) practice organisation; 3) links with other services including with community pharmacies; and 4) individuals’ everyday lives (see Tables 1 and 2). This qualitative study adhered to the qualitative research guidelines (RATS) [19].

**Table 1:** Interview schedule for clinicians

NPT construct	Questions
Coherence	• What are your initial thoughts?
Coherence	• How would you use it?
Cognitive Participation	• How does it fit with your current practice? What considerations of renal function do you give during episodes of acute illness for people with CKD?
Cognitive Participation	• Do you consider temporarily stopping certain medications during times of acute illness with patients with CKD?
Cognitive Participation	• What might influence it becoming a routine part of practice?
Collective Action	• Do you discuss with patient with CKD during their review appointments how they should manage an episode of acute illness?
Collective Action	• How does this change when people have multiple conditions?
Coherence	• How do you manage patients with coronary heart failure to prevent AKI? What would influence your decision for recommending patients to stop ACE inhibitors or ARBs or NSAIDS?
Coherence	• Can you give me some examples of when you have temporarily stopped medications for patients with other conditions? What are the implications in the practice? How did you remind patients when they had to start taking the tablets again?
Cognitive Participation	• How will you identify the most vulnerable 2 %? What is the focus of their care for you (eg unplanned admissions) and what will that entail?
Collective Action	• How would the action plan fit with your plans for supporting the most vulnerable 2 %?
Collective Action	• How do you co-ordinate with hospital staff about medications people are taking and their conditions?
Collective Action	• What happens when someone is discharged from hospital? What information are you given and who is this from?
Collective Action	• How is this co-ordinated between the hospital and your practice/pharmacy? How do you co-ordinate this with the local pharmacists/GPs?
Reflexive Monitoring	• What are some of the logistical issues in managing changes to medications?
Collective Action	• How do you co-ordinate changes in medicines? What are the implications in the work that you have to do to manage it and to restart medicines?
Collective Action	• What other services do you co-ordinate with to manage patients with acute illnesses? (eg community matrons and district nursing team)

**Table 2:** Interview schedule for patients

NPT construct	Questions
Coherence	• What do you think of the action plan?
Coherence	• If you imagine you had to use it: can we talk through the process that you would go through?
Cognitive Participation	• Would you stop temporarily tablets if they were to manage pain? A heart condition? What would influence you in making that decision?
Collective Action	• Who do you think should give you this?
Collective Action	• Who do you think would support you to stop tablets for a short period?
Collective Action	• Would you want to be reminded at the end of the tablet break that you should start taking them again?
Coherence	• Would you use it?
Collective Action	• What happens during review appointments with GPs and practice nurses?
Coherence	• Have you discussed what you should do if you start to feel unwell eg flu?
Coherence	• What do you do when you’re starting to feel unwell?
Coherence	• When do you go to see your doctor/nurse?
Cognitive Participation	• Do you currently take any medications?
Cognitive Participation	• Do you have a system for managing them?
Coherence	• Have you ever had to stop taking any of your tablets for a short time because you were ill?
Coherence	• Were you given any information (written or verbal)?
Collective action	• What happens when you visit community pharmacists?

### Data analysis

Two researchers read the transcripts independently (RM and TB) and through comparative analysis independently noted down main themes and sub-themes that emerged. Key themes were identified through discussion between RM and TB. Key themes and quotes were circulated to all the authors for comments and discussion. A final set of themes and sub-themes were agreed by all authors. Atlas ti 7.5 qualitative analysis software was used to support analysis. NPT was used within the analysis as a sensitising concept to the work involved in implementation across primary care [17, 18]. NPT has four main underlying constructs that influence implementation: coherence (i.e. meaning and sense making by the participants); cognitive participation (i.e. engagement and commitment by the participants); collective action (i.e. the work needed to make the intervention function); and reflexive monitoring (i.e. reflection and appraisal of the intervention by participants) [20]. We used NPT to create a framework to examine the context of implementation and combined this with a thematic approach to examine and refine emerging themes [21]. Analysis examined all the data across and within each setting using our research aims.

This study was given ethical approval by Research Ethics Committee North West-Preston reference 14/NW/0099.

## Results

Twelve GPs, 8 Practice Nurses, 12 Pharmacists and 10 patients were interviewed (see Table 3). In general, participants tended to express initial enthusiasm and understanding surrounding the rationale for sick day rules to prevent AKI. Sick day rules were seen to fit with the delivery of comprehensive care.

**Table 3:** Participant characteristics

	Number
Age (years)	
20–40	9
41–50	12
51–60	9
60+	10
Gender	
Male	20
Female	20
Patients	10
Number of long term conditions	
1	1
2–4	4
5+	5
General Practitioners	12
Years of experience	
1–5	2
6–15	2
16+	8
Practice nurse	8
Years of experience	
1–5	1
6–15	1
16+	6
Community Pharmacists	10
Years of experience	
1–5	3
6–15	1
16+	6

*I’d use it with my patients who are identified as CKD, who are on an ACE or an ARB…I don’t think there’s any reason not to give all of them this, the same way as you would a diabetic patient, you know, the sick day rules for their insulin*

ID04 (Practice Nurse)

Patient participants also described an initial engagement with the idea of temporary cessation of medicines if directed by a healthcare professional to stop the medicines and an explanation of why there would be a change in their previous response to these acute illnesses. Patient participants also raised uncertainty about whether they would be able to distinguish between the symptoms of different conditions which may affect their recognition of when to begin temporary cessation.

*It’s never happened in the past. I’ve never been advised to stop taking them but if the doctor advised that, I’d stop taking them…Listen, if the doctor tells me that I need to take a tablet I take it. If he says, stop, I stop. I have faith in the man… I have flu vaccinations so I don’t often get flu that I’m aware of and I don’t actually go to the doctor with it…if I’ve had the flu, if it was flu, I’ve just carried on… I’m not sure whether, you know, you just think it’s a bad cold. It could be flu, but it might just be a bad cold, so I don’t know, but if I was to go to the doctor and he said, you’ve got flu, stop taking x, y and z, I’d stop taking them.*

ID12 (Patient)

However, during the course of interviews, initial interest tended to be diminished by a range of factors influencing implementation. Factors affecting the uptake and use of sick day rules related to collective engagement within and across services, uncertainties around embedding sick day rules into existing clinical care, as well as resourcing the integration of AKI initiatives into routine practice.

### Collective engagement with sick day rules

It was considered an essential requirement by patients and healthcare professionals that a consistent message about temporary medicine cessation and AKI prevention was given by all health care professionals.

*Everybody pulling together and giving out the same message, including the receptionists. Everybody…It will be good practice every time you see them to say, and you do have your action plan, or you do have your sick day rules.*

ID04 (Advanced Nurse Practitioner)

From a community pharmacy perspective it was seen as important that the implementation of sick day rules did not disrupt working relationships with local GPs.

*The GPs are trying to make sure people do come to us…I’d rather we be able to do something with the blessing of the local surgeries than sending them back, I think they wouldn’t want that.*

ID14 (Pharmacist)

In order to engage, pharmacists needed to know that GPs were on board with this initiative, with a preference expressed for adopting a secondary reminder role that was more congruent with their existing remit.

*I think it’s a really good idea yeah, and as pharmacists we’d be happy to remind patients like we do with don’t take your statin things*

ID15 (Pharmacist)

Boundaries of responsibility (both legal and professional) were highlighted in terms of having a role in discussing the temporary cessation of medicines. These boundaries were reinforced by limits to professional education and training. As with community pharmacists, practice nurses also needed to ensure GP engagement in order to have confidence to implement sick day rules into their clinical practice.

*I would consult with the doctor and let him make the decision. I would never do it independently…Because I wouldn’t feel competent, or confident…no court in the land would support me. It would be very wrong and very unprofessional*.

ID01 (Practice Nurse)

Patients also expressed increased confidence in medicines management when there was evidence of common agreement with professionals involved in their care. Patient accounts depicted the need for their care providers to have access to their comprehensive information to make informed clinical recommendations. Whilst this was not always considered to have to be the GP, the GP role as the link between primary care providers and consultants gave patients’ confidence that they would have access to all necessary information to ensure their safety.

*One of the things that my GP thinks about doing…he will make sure that he gets confirmation from the renal consultant that it’s okay to do it. They’re very wary of prescribing for me… There’s a communication between the two.*

ID35 (Patient)

In practices that had experience of using sick day rules, co-ordination across services was considered important when considering the implementation of AKI initiatives. Simply giving the sick day rule plan on its own tended to be seen as insufficient by clinicians who had implemented a plan.

*We didn’t because we did it all in-house, but that would’ve been another way we could’ve done it through the local pharmacy….we had a list and we had all these leaflets and we wanted to get them out as quickly as possible. But yes, I mean in retrospect involving the local pharmacist would’ve been a good idea.*

ID26 (GP)

In order to have confidence to implement sick day rules to prevent AKI, participants’ accounts highlighted the need for wider, collective, commitment with the initiative and that clarity in roles and responsibilities was central to this process.

*Yes, I think the community matrons are a resource that could really do this. Them, the heart failure nurses as well, so I think there’s a lot within the community who could…I mean, we’re meant to tie all this stuff together…And then, there’s the nursing home staff…the problem is it will escalate because no one wants to take responsibility, and the buck’s got to stop somewhere.*

ID03 (GP)

Patient participants also identified the need for supported approach to temporary cessation where they were able to contact their GP if they had any questions and the action plan acted as an aide memoire that would have to be stored in accessible place, such as with their medicines.

*The problem is, as you get older your memory isn’t great, it’s hard to remember to do things, especially if you’re not well…I would keep it with my medications so that I’m, you know, I have a special drawer that I keep all my pills, which unfortunately are quite a lot. And I would keep it there; you know, fully filled in and perhaps have my doctor’s contact number on it as well.*

ID20 (patient)

### Embedding sick day rules into clinical encounters

Whilst recognising that sick day rules were not currently part of routine practice, health professionals suggested that they may be relevant for a range of patients. Participants suggested that sick day rules could be implemented in review appointments. Episodes of acute illness were also considered an ideal opportunity to remind patients of sick day rules, with IT systems prompting reminders.

*We are entirely reliant on the computer, so as soon as you entered any patient’s notes, if they were one of those, it came up with a prompt that you had to press a button to get rid of, so you couldn’t really avoid it.*

ID28 (GP)

Community pharmacists identified opportunities they had for reminding patients about AKI prevention as they dispensed medicines. They also highlighted their limited access to diagnostic information (such as knowledge of a patient’s kidney function), which was seen to constrain their potential to optimise conversations with patients.

*I think as pharmacists we could deliver it in a positive way because we’d have the time to sit with the patient, do a medicines review, or even without a medicines review the fact is that when we’re dispensing any medication you’ve got time to really engage with them, probably more so than the GP would… But identifying the patients in the first place would be a big stumbling block for us.*

ID18 (Pharmacist)

Some patient participants similarly described a more limited confidence in the advice given by the pharmacists as they did not necessarily have access to patient information.

*I:..who it should come from, is it the GP or would it be the nurse or the pharmacist?*

*R: GP or nurse…I suppose a GP or the nurse at the GP centre would know more about my history than the pharmacist would, so I would be more likely to take their word for it.*

ID38 (Patient)

Practice nurse accounts varied in terms of the ease with which they may implement sick day rules within their clinical work. For some, implementation was considered relatively straight forward as patients already attended review consultations with an existing expectation that education and support would be provided.

*For all the patients who would be coming in to me for the reviews, for myself to do it I wouldn’t see it as a problem… because we’re talking about a lot of things with them, I do feel it’s not as though it’s going to take me another 20 min to go through … it’s quite self-explanatory. You’d be documenting the medicines… I could use it.*

ID09 (Practice Nurse)

However, for other practice nurses, there was uncertainty on how to embed sick day rules without creating additional workload. Though participants considered kidney function as part of a comprehensive approach to care, it was not necessarily clear how to integrate AKI initiatives into existing practices given other clinical priorities and patient safety issues.

*You look at them holistically… It’s not just the flu. You’ve got to look at everything else that’s going on…you get patients obviously with multiple medical problems and you have to try and remember to include everything in your consultation. It’s sometimes quite hard.*

ID01 (Practice Nurse)

Similarly, some GPs reflected a sense of workload pressure and the challenge in determining which information and guidance to give precedence.

*I know as a GP I don’t know everything but I know that when problems arise I need to start tackling them, and how do I tackle them really? …I mean general practice is to do with assessing risk…when new information comes in it’s assessing the need and the risk of that and how quickly I need to do it*

ID13 (GP)

Whilst deemed important, few GPs who had not used a sick day rule plan described currently discussing kidney care with patients in the context of preventing AKI during episodes of acute illness.

*We’re pretty good now at telling people how to take their ACEs. We’re not so good at telling people to stop…when people are unwell, I think that that slips our mind… the majority of these illnesses may well start off the majority of times be self-limiting, they don’t get to us… But then, that’s predisposing them to develop other things and progress to having acute kidney injury, whereas if they do know to stop them each time then that’s much better.*

ID03 (GP)

Again, across clinician interviews, professional responsibility for managing risk was seen as a key driver of clinical care. In this context, they described trade-offs and uncertainties when considering kidney function in the context of clinical management of patients with complex co-morbidities including heart failure.

*You look at what effect, you know, any diuretics they’re having… What’s their blood pressure doing, because if their blood pressure has plummeted as well then, yes, it can make sense to stop things, so it’s weighing up all of these different things*.

ID03 (GP)

### Resourcing implementation of sick day rules

Capacity within primary care to maintain the potential level of work required was raised by the majority of clinician participants. Linking the implementation of sick day rules to existing incentives structures (such as the chronic illness review or the Medicine Use Reviews (MUR) [22]) were mechanisms identified to support this additional work. However, whilst it was seen as a small amount of work with each individual, the cumulative effect was deemed inhibitive.

*If they were on medication, we’d just stick a pharmacy sticker on the back, highlight it, and then we’d go out and have a five minute chat to patients… although we’d have a lot…the only thing that I worry about then is having to do a lot of five minute chats and the workload.*

ID15 (Pharmacist)

A major limitation to the implementation of sick day rules to prevent AKI was how to support patients who used dosette boxes. The additional work involved in identifying, removing and resealing the tablets was considered to be too much unless remunerated.

*What we would do is help them identify which ones they need to not take…we don’t interfere with blister packs once they go out. So we wouldn’t be prepared to take them out and reseal them because then that’s more unremunerated work, …If they’re reissued it’s fine, we just redo them…from new stock, and that’s fine, but that’s part of the politics of this whole area really.*

ID30 (Pharmacist)

Participants highlighted the roles of other support staff that may need to be resourced in order to successfully implement the process of temporary cessation. This included costing the potential increase in workload for pharmacy delivery drivers associated with changing prescriptions and managing the dosette boxes.

*What the GP might phone and say I want to take this patient, well we get phone calls like that anyway that doctors quite often do the home visits and changing medications… if this is something that’s…rolled out, we will be doing a lot of patients that have, especially around winter time as well when a lot of these elderly patients are getting flu…we may need to employ another driver to, you know…*

ID15 (Pharmacist)

In general practice this additional workload could also have implications for receptionists and clinicians.

*It would need a telephone consultation to make sure that the patient has restarted their medication, because like I said, people will stop, it’s remembering to get started on it again. …I think we probably would get a lot of phone-calls, and they would obviously go through to reception and not to us. So that might be a bit of an issue.*

ID23 (Practice Nurse)

Explaining the purpose of sick day rules was of particular importance because of the potential change to patients’ normal acute illness response and expectations of care provision.

*It would just be absolutely part of what we said before. When you put them on it, you say, you know, I know the media says don’t come in with a cold or flu, but in your case it’s a little bit different. You’re older, your kidneys aren’t working as well as they used to.*

ID04 (Advanced Nurse Practitioner)

Finally, a sense of fatigue surrounding the changing nature of financial incentivisation of primary care was identified as a factor that might influence their implementation.

*I think there’s huge pressure at the moment…Every year, there’s a whole new set of rules. Whether it be QOF or LESs and DESs, which require very little that directly benefits patients…I think there was a great fatigue about that. And there was virtually nothing had come up that would ever improve the quality of patient care obviously, like this would*

ID11 (GP)

## Discussion

The study analysis highlighted tensions between the prevention of AKI as a fundamental component of safe care and the reality of embedding it within routine practice. The study analysis suggested a common understanding (coherence [18]) concerning the rationale for sick day rules to prevent AKI. However, in order to have confidence to engage with their implementation, participants’ accounts highlighted a need for clarity of roles and responsibilities within and across services. Professionals’ capacity to undertake the work surrounding temporary cessation was deemed limited unless adequately resourced.

Central to current clinical recommendations is the identification of risk factors (e.g. taking certain medications during acute illness) and their temporary removal [12] but participant accounts stressed the complexities of this in practice. In particular, relationships between professionals across primary care had the potential to support or undermine implementation. The micro level systems of General Practices, or Pharmacies, influenced implementation and was shaped by concerns about capacity to undertake additional work, variable access to patients’ diagnostic information and the cumulative effect of processes to work (such as reissuing dosette boxes) that would need to be undertaken. At a macro level, the organisational context that was needed to support implementation was a need to have clear guidance outlining boundaries of responsibility for giving recommendations.

Systematic reviews of self-management for patients with chronic obstructive pulmonary disease indicate action plans to support the management of exacerbations are only likely to have a positive effect on quality of life and health care utilization if delivered as part of a multifaceted programme [23–25]. Key themes that emerged from this study also suggest that sick day rules alone are unlikely to have effect on the prevention of AKI without resourcing wider strategies to support implementation (including support to address the issue of responsibility across professions).

AKI prevention was considered to respond to a clinical need rather a policy driven approach. Patients’ prioritisation of conditions may also influence the adoption of prevention strategies. Prioritisation of conditions changes over time [26] and may be influenced by the timing of information provision and education around diagnosis. In this context, care plans (such as sick day rules) may be a tool to support the discussion of AKI prevention and renal function.

In terms of implementing sick day guidance, health professional concerns may reflect current limited evidence surrounding the temporary cessation of medicines during episodes of acute illness [27]. Though recommended in clinical guidance [8, 12] hypotheses also exist that continuation of ACE Inhibitors may result in a reduction in glomerular filtration (reflected in Serum Creatinine rise) but have a protective effect on renal tubular function [28, 29]. As such a large scale evaluation measuring all-cause mortality or any benefits and harms of sick day rule use in different patient populations is needed to determine clinical and cost-effectiveness.

Participants’ accounts illuminate the distributed nature of work surrounding the implementation of NICE guidance regarding temporary cessation of medicines for AKI prevention. Integrating initiatives to prevent AKI within existing funding structures may help resource additional work identified. For example, the New Medicine Service (NMS) [25] as well as the MUR [22] may support dialogue within community pharmacy. Aligning key policy initiatives is a necessary step. In April 2014, NHS England launched the National AKI Programme [9]. At the same time, the Unplanned Admissions Enhanced Services was introduced in which GPs are funded to identify and proactively case manage patients deemed most at risk of emergency hospitalization [30]. Research evaluating the incorporation of targeted actions plans to prevent AKI compared with the use of more generic care plans to avoid unplanned hospitalization may also be warranted [29, 31]. NPT helped to illuminate the context and localised systems approach that may need to be adopted to work with local stakeholders to implement sick day guidance. In essence, this implementation should include a collective space for different health professionals to meet and discuss ongoing implementation, and to address issues of communication and clarity of roles and responsibilities to ensure engagement and participation by all stakeholders.

### Strengths and limitations

This study draws on both prospective accounts of GP, Practice Nurse, Community Pharmacist and patient accounts about the potential enablers and constrainers of implementation as well as retrospective accounts of those who have already implemented a sick day rule plan. This is a strength of this study as it supports an understanding of the processes of implementation across different settings. As AKI prevention is a national patient safety priority, there is a need to understand these processes and to recognise the realities of everyday practice and management to optimise the implementation of AKI prevention strategies that supports both patients and clinicians. Use of NPT as a sensitizing tool [18] helped illuminate different types of work (e.g. co-ordination between professionals (i.e. collective action) or engagement with specific tasks or responsibilities (i.e. cognitive participation)) surrounding the implementation of sick day rules. One limitation is that in practices that had already implemented a sick day rule plan, there were no data about changes to rates in community-acquired AKI following implementation. Whilst this will be important for future studies, this study was focused on examining the processes of implementation this enabled us to understand how sick day rules were embedded, or not, within different local primary care systems. Understanding these processes of implementation is important for developing future national interventions. Further research in different settings (such as nursing homes) is needed and exploration of a wider range of patient and carer experience concerning the use of sick day rules in everyday life, such as during an acute illness episode.

## Conclusions

This study sought to understand the implementation of sick day rules to prevent AKI in primary care. Although seen to fit with the delivery of comprehensive care, the findings highlight the need for shared understanding of roles and responsibilities in order to become part of routine practice. Consideration should be given to resourcing AKI initiatives through integration into existing funding mechanisms as well as developing an evidence base concerning their effectiveness.

## Abbreviations

ACE, angiotensin-converting-enzyme; AKI, Acute Kidney Injury; ARB, Angiotensin Receptor Blocker; CKD, chronic kidney disease; COPD, chronic obstructive pulmonary disease; GP, General Practitioner; IT, information technology; MUR, Medicine Use Review; NHS, National Health Service; NICE, National Institute for Health and Care Excellence; NMS, New Medicine Service; NPT, normalization process theory; QOF, quality outcomes framework; UK, United Kingdom

## Additional file

Additional file 1: A ‘sick day rule’ plan for AKI prevention. (DOCX 292 kb)
