# Supplementary material for: Preventing Acute Kidney Injury: a qualitative study exploring ‘sick day rules’ implementation in primary care
Source: BMC Fam Pract. 2016 Jul 22;17:91. doi: 10.1186/s12875-016-0480-5 (PMC4957384; doi:10.1186/s12875-016-0480-5)
Supplement: Additional file 1: — A ‘sick day rule’ plan for AKI prevention. (DOCX 292 kb) [file 12875_2016_480_MOESM1_ESM.docx]

A ‘sick day rule’ plan for AKI prevention^a^

^a^ Final version of Sick day rule plan/booklet which was informed by Patient and Public Involvement collaborators, feedback from participants and other AKI prevention initiatives developed by: Royal Derby and Cornwall Hospitals; Highland Sick Day rules; Wrexham Maelor Hospital; and Dr John Harty, Southern Health and Social Care Trust, Northern Ireland
